# Rhinovirus C Infection Induces Type 2 Innate Lymphoid Cell Expansion and Eosinophilic Airway Inflammation

**DOI:** 10.3389/fimmu.2021.649520

**Published:** 2021-04-22

**Authors:** Charu Rajput, Mingyuan Han, Tomoko Ishikawa, Jing Lei, Adam M. Goldsmith, Seyedehzarifeh Jazaeri, Claudia C. Stroupe, J. Kelley Bentley, Marc B. Hershenson

**Affiliations:** Department of Pediatrics, University of Michigan Medical School, Ann Arbor, MI, United States

**Keywords:** asthma, rhinovirus, innate cytokine, viral infection, exacerbation, ILC2

## Abstract

Rhinovirus C (RV-C) infection is associated with severe asthma exacerbations. Since type 2 inflammation is an important disease mechanism in asthma, we hypothesized that RV-C infection, in contrast to RV-A, preferentially stimulates type 2 inflammation, leading to exacerbated eosinophilic inflammation. To test this, we developed a mouse model of RV-C15 airways disease. RV-C15 was generated from the full-length cDNA clone and grown in HeLa-E8 cells expressing human CDHR3. BALB/c mice were inoculated intranasally with 5 x 10^6^ ePFU RV-C15, RV-A1B or sham. Mice inoculated with RV-C15 showed lung viral titers of 1 x 10^5^ TCID_50_ units 24 h after infection, with levels declining thereafter. IFN-α, β, γ and λ2 mRNAs peaked 24-72 hrs post-infection. Immunofluorescence verified colocalization of RV-C15, CDHR3 and acetyl-α-tubulin in mouse ciliated airway epithelial cells. Compared to RV-A1B, mice infected with RV-C15 demonstrated higher bronchoalveolar eosinophils, mRNA expression of IL-5, IL-13, IL-25, Muc5ac and Gob5/Clca, protein production of IL-5, IL-13, IL-25, IL-33 and TSLP, and expansion of type 2 innate lymphoid cells. Analogous results were found in mice treated with house dust mite before infection, including increased airway responsiveness. In contrast to *Rora* ^fl/fl^ littermates, RV-C-infected *Rora* ^fl/fl^ *Il7r* ^cre^ mice deficient in ILC2s failed to show eosinophilic inflammation or mRNA expression of IL-13, Muc5ac and Muc5b. We conclude that, compared to RV-A1B, RV-C15 infection induces ILC2-dependent type 2 airway inflammation, providing insight into the mechanism of RV-C-induced asthma exacerbations.

## Introduction

First reported in 2006 (1, 2), rhinovirus C (RV-C)^4^ has been associated with severe respiratory illnesses in children and adults, often requiring hospitalization (3–15). Infections with RV-C are more likely to occur in children with a history of asthma or who develop asthma (6, 10–14). In addition, compared to RV-A, children with RV-C have been reported to have severe lower respiratory tract infections including wheezing, oxygen supplementation and intensive care unit admission (7, 8, 10, 11, 15).

Despite increasing recognition of RV-C as a cause of severe exacerbation, little is known about the pathogenesis of RV-C infections. Thus far, 55 RV-C genotypes have been reported which are believed to be synonymous with serotypes (16, 17). In contrast to major and minor group RV-A and RV-B viruses, RV-C does not utilize intercellular adhesion molecule (ICAM)-1 or low density lipoprotein family receptors (LDL-R). This is likely due to the fact that the hydrophobic pocket in VP1 is filled with multiple bulky residues (18). Instead, RV-C utilizes cadherin related family member 3 (CDHR3) as a receptor (19). Individuals with CDHR3 C529Y variants (AG and AA genotype) appear to be more susceptible to RV-C infection, as this variant is localized on the airway epithelial cell surface, where it is accessible to viral infection, in contrast to the more common GG genotype which is mostly localized to the cytoplasm (19–21).

RV-C has been refractory to study in part because it is difficult to grow *in vitro.* RV-C has been grown in primary mucociliary-differentiated human airway epithelial cells grown at air-liquid interface (22, 23) and HeLa cells transduced with the CDHR3 AA allele (HeLa-E8 cells) (24).

The mechanisms by which RV-C promotes severe respiratory illness are unknown. Since type 2 inflammation is an important disease mechanism in a large subgroup of individuals with asthma [reviewed in (25)], we hypothesized that RV-C infection, in contrast to RV-A infection, preferentially stimulates type 2 inflammation, leading to exacerbated eosinophilic inflammation. Mouse models have been utilized to study the host response against respiratory enteroviruses such as major group RV-A16 (26), minor group RV-A1B (26, 27) and enterovirus D68 (28). To study underlying mechanisms, we obtained cDNA encoding RV-C15 and HeLa-E8 cells from James Gern and Yury Bochkov (University of Wisconsin). We inoculated mature BALB/c mice with RV-C15, comparing inflammatory responses to those induced by RV-A1B. In addition, we compared the response of allergen-sensitized and challenged mice to the two viruses.

## Material and Methods

### Generation of RV-C15 and RV-A1B

**Figure 1:**
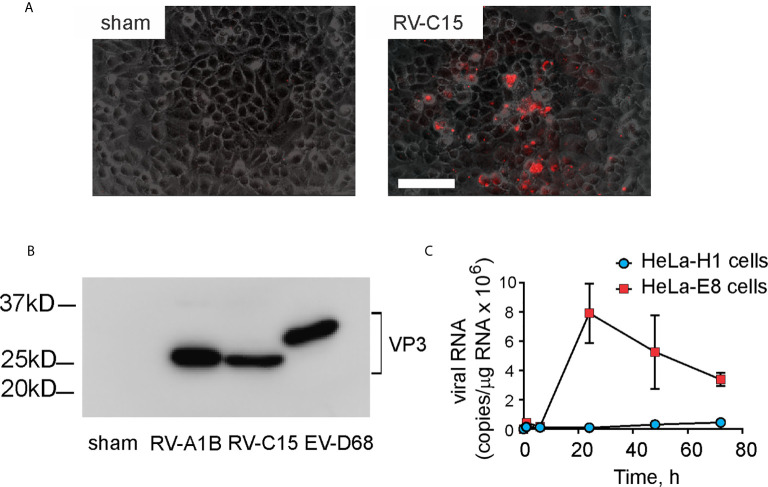
Generation of RV-C15. Full-length cDNA encoding RV-C15 was reverse transcribed, and the resulting vRNA transfected into HeLa-H1 cells. Virus was harvested from the cell supernatants and used to infect HeLa-H1 cells or HeLa-E8 cells expressing human CDHR3 C529Y. **(A)** RV-C15 induces cytopathic effects on HeLa-E8 cells. Areas of cell damage matched staining with AlexaFluor-labeled anti-VP3. The white bar is 100 µ **(B)** Anti-VP3 immunoblots of HeLa cell lysates infected with RV-A1B, RV-C15 and enterovirus D-68. Concentrated viral preparations were resolved by SDS-PAGE and probed with anti-VP3. **(C)** Infection of Hela-E8 and HeLa-H1 cells with RV-C15. RV copy number was determined by qPCR. Data shown are mean ± SD, *n* =3.

Full length cDNA encoding RV-C15 and HeLa-E8 stable cells expressing human CDHR3 C529Y (19) were provided by James Gern and Yury Bochkov, University of Wisconsin. The cDNA was reverse transcribed and resulting full-length vRNA transfected into HeLa-H1 cells (ATCC, Manassas, VA) using lipofectamine (ThermoFisher Scientific, Waltham, MA). Virus was harvested from the HeLa-H1 cell supernatants and used to infect HeLa-E8 cells. Initial RV-C15 from transfected HeLa-H1 cells does not cause obvious cytopathic effects or form plaques in HeLa-E8 cells. However, upon close inspection, areas of cellular damage matched staining with Alexa Fluor 555-conjugated anti-mouse EV-D68 VP3 (**Figure 1A**). Anti EV-D68 VP3 (GeneTex, Irvine, CA) recognizes VP3 from EV-D68, RV-A1B and RV-C15 (**Figure 1B**). RV-C15 replicated in HeLa-E8 cells but not HeLa-H1 cells (**Figure 1C**). RV-A1B (ATCC), a minor group virus that infects mouse cells (29) was grown in Hela-H1 cells. The two viruses were propagated, concentrated and partially purified from infected HeLa cell lysates by means of ultrafiltration with a 100-kDa cutoff filter assay, as described previously (27). Similarly concentrated and purified HeLa cell lysates were used for sham infection. With propagation and concentration needed to produce sufficient quantities for animal experiments, we observed amplified cytopathic effects in RV-C15-infected HeLa-E8 cells. We took advantage of this result to test for live virus in the lungs of infected mice (see Results below).

### RV-C15 Infection and Treatment

All animal usage was approved by the Institutional Animal Care and Use Committee of University of Michigan and performed under National Institutes of Health guidelines. Eight-to-twelve-week-old female BALB/c mice (Jackson Laboratories, Bar Harbor, ME) were inoculated with 5 x 10^6^ PFU equivalents (ePFU) of RV-C15 in 50 µL PBS by intranasal instillation. RV-C ePFU was calculated based on a calibrated standard curve for RV-A1B (18). Additional mice were inoculated with 5 x 10^6^ ePFU RV-A1B (in 50 µL PBS) or an equal volume of sham HeLa cell lysate. In additional experiments, we examined RV-C15-induced mRNA expression in *Rora* ^fl/fl^ *Il7r* ^Cre^ mice and *Rora* ^fl/fl^ littermates (from Dr. Andrew McKenzie, MRC Laboratory of Molecular Biology, Cambridge, UK). Based on the requirement of RORα for ILC2 development (30), *Rora* ^fl/fl^ *Il7r* ^Cre^ mice are ILC2 deficient (31). *Il7r* ^Cre^ mice were originally generated by Dr. Hans-Reimer Rodewald (Division of Cellular Immunology, German Cancer Research Center, Heidelberg) (32).

Using specific forward and reverse primers, RNA from passaged virus was used to produce random primed first strand cDNA and overlapping products using Phusion high fidelity DNA polymerase (New England Biolabs, Ipswich, MA) of about 2 kB each. These products were agarose gel purified and processed for Sanger sequencing (University of Michigan DNA Sequencing Core). Primers used for PCR production and sequencing are shown in the **Supplemental Table**. Sequencing through nucleotide 7042 of the W-10 RV-C15 reference sequence GU219984, we did not detect the previously described mutations in VP1 or 3A (24).

### Model of Allergic Airways Disease

BALB/c mice were sensitized through the intranasal route with 100 μg *D. pteronyssinus* house dust mite (HDM) extract in 50 μl PBS (Greer Labs, Lenoir, NC) by intranasal installation on day 1 and challenged with 10 μg HDM on days 11 and 12 (33). On day 13, mice were inoculated through the intranasal route under Forane anesthesia with RV-C15, RV-A1B or sham, as noted above.

### Real-Time Quantitative PCR

Lungs were harvested at different points and RNA was extracted with Trizol (Invitrogen, Carlsbad, CA). Lung RNA was isolated using an RNAeasy kit (Qiagen). cDNA was synthesized from 2 μg of RNA using high capacity cDNA synthases kit (Applied Biosystems, Foster City, CA) and subjected to quantitative real-time PCR using specific primers for mRNA (**Table 1**). The level of gene expression for each sample was normalized to GAPDH unless otherwise specified. RV copy number (vRNA) was determined by qPCR using previously published primers (34).

### Generation of a Peptide Directed Anti-CDHR3 Antibody

The CDHR3 protein has extracellular calcium binding domains and a sialic-acid modified Asn186 important for RV-C15 binding (19). Hopp-Wood hydrophilicity analysis (DNASTAR, Madison WI) of the NIH BLAST sequence alignments of NP_689963.2 (human) and NP_001019649.1 (mouse) revealed a short conserved peptide (human amino acids 154-167, YQVEAFDPEDTSRN) in the second calcium binding domain representing a possible selective antigen for both human and mouse CDHR3. A rabbit polyclonal antibody was produced and purified using affinity chromatography (Genscript, Piscataway, NJ).

HeLa-H1 cells, HeLa-E8 cells, and mouse lungs were lysed, cellular proteins resolved by 10% SDS-PAGE, and proteins transferred to a nitrocellulose membrane. Membranes were probed with anti-CDHR3. Signals were amplified and visualized with horseradish peroxidase-conjugated secondary antibody (BioRad, Hercules, CA) and chemiluminescence solution (Pierce, Rockford, IL). To determine the specificity of the observed bands, primary antibody was incubated with cysteine-conjugated YQVEAFDPEDTSRN peptide. Anti-CDHR3 (1 µg/mL) recognized 100 kD bands in CDHR3-expressing HeLa-E8 cells and mouse lung lysate but not HeLa-H1 cells (**Supplemental Figure 1A**). Recognition of these bands was abolished by addition of YQVEAFDPEDTSRN peptide (10 µg/mL). Other bands, perhaps representing proteins with calcium binding domains, remained. HeLa-E8 cells, but not HeLa-H1 cells on glass coverslips stained positively with AF488-conjugated anti-CDHR3. Staining was blocked with addition of either unlabeled rabbit IgG or YQVEAFDPEDTSRN (**Supplemental Figure 1B**).

### Lung Histology and Immunofluorescence

Lungs were harvested at different points, fixed with 10% formaldehyde overnight and paraffin embedded. Blocks were sectioned at 500 μm intervals at a thickness of 5 μm, and each section was deparaffinized, hydrated and stained. To visualize inflammation, sections were stained with H&E. Other lung sections were incubated with anti-CDHR3, anti-EV-D68 VP3, mouse anti-acetyl α-tubulin (MilliporeSigma, Burlington, MA), rat anti-IL-25 (Biolegend), goat anti-IL-33 (eBioscience), rat anti-TSLP (Biolegend), rat anti-CD123 (Biolegend) or isotypic IgG. Antibodies and IgGs were labeled with AlexaFluor NHS esters (ThermoFisher) according to manufacturer’s instructions. ILC2s were identified as IL-13- and GATA3-positive, and T cells were identified as IL-13-, GATA3- and CD3-positive. Images were visualized using an ApoTome microscope (Carl Zeiss, Thornwood, NY) or a Leica SP5 inverted laser confocal microscope (Buffalo Grove, IL).

### Flow Cytometric Analysis

Lungs were harvested 2 days after sham, RV-C15 and RV-A1B treatment. Lungs were perfused with PBS containing EDTA and minced and digested in collagenase IV. Cells were filtered and washed with red blood cell lysis buffer, and dead cells were stained with Pacific Blue Live/Dead fixable dead staining dye (Invitrogen). Nonspecific binding was blocked by 10% FBS with 1% LPS-free BSA and 5-µg rat anti-mouse CD16/32 (BioLegend, San Diego, CA) added. To identify ILC2s, cells were then stained with FITC-conjugated antibodies for lineage markers [CD3ϵ, T-cell receptor- β (TCR β), B220/CD45R, Ter-119, Gr-1/Ly-6G/Ly-6C, CD11b, CD11c, F4/80, and FcϵRIα; all from BioLegend], anti-CD25-peridinin- chlorophyll-protein complex (PerCP)-Cy5.5 (eBioscience), anti-CD127-allophycocyanin (APC; eBioscience) and anti-ST2-phycoerythrin (PE)-Cy7 (BioLegend), as described (35). Cells were fixed, subjected to flow cytometry, and analyzed on a LSR Fortessa (BD Biosciences, San Jose, CA). Positive/negative staining was determined using fluorescence minus one (FMO) controls. Data were collected using FACSDiva software (BD Biosciences) and analyzed using FlowJo software (Tree Star, Ashland, OR).

### Bronchoalveolar Lavage

**B**ronchoalveolar lavage (BAL) was performed using 1 ml PBS aliquots. Cytospins were stained with Diff-Quick (Dade Behring, Newark, DE) and differential counts determined from 200 cells (36).

### ELISA

Mouse lungs were harvested, homogenized in PBS plus Roche Complete Protease Inhibitors (MilliporeSigma), and snap frozen in liquid nitrogen. After thawing and resuspension at 4°C, particles were centrifuged at 10,000 x *g* for 30 min, and the supernatant was diluted serially in the homogenization buffer for ELISA of IFN-β, IFN-λ, IL-5, IL-13, IL-25, IL-33 and TSLP according to the manufacturer’s instructions (R&D Systems, Minneapolis, MN and eBioscience).

### Measurement of Airway Responsiveness

Mice were anesthetized, intubated, and ventilated with a Buxco FinePointe System (Wilmington, NC). Mice were administered nebulized saline and increasing doses of nebulized methacholine to assess airways responsiveness (27).

### Airway Epithelial Cell Culture

Mouse airway epithelial cells were purchased from Cell Biologics (Chicago, IL). Primary airway epithelial cells were cultured in Transwells at air-liquid interface as described previously, with some modifications (37). Briefly, airway epithelial cells were cultured under submerged conditions in complete PneumaCult-Ex Plus medium (Stemcell Technologies, Vancouver, CA) for 1 week. Cells were transferred to Transwells and cultured with complete medium in both basal and apical wells until confluence was reached. Cells were then maintained at air-liquid interface for three weeks in PneumaCult-ALI maintenance medium. Cells were infected with sham, RV-C15 or RV-A39 at a multiplicity of infection (MOI) of 2 for 12 hrs. RV-A39 was purchased from ATCC and purified from infected HeLa-H1 cell lysates by ultrafiltration with a 100-kDa cutoff filter. Selected cell cultures were fixed and stained with AlexaFluor 488-conjugated anti-acetyl α-tubulin (MilliporeSigma, Burlington, MA) and AlexaFluor 555-conjugated anti-VP3. Immunoreactivity was visualized with a NikonA1 laser confocal microscope.

### Data Analysis

Normality was tested using the Shapiro-Wilk test. Group mean data are represented as mean ± SEM or median ± interquartile range as appropriate. Statistical significance was assessed by unpaired t-test, Mann-Whitney test, one-way analysis of variance (ANOVA) or Kruskal-Wallis test as appropriate. Group differences were pinpointed by the Tukey or Dunn’s multiple comparison test.

## Results

### RV-C15 Persists in Lungs at Similar Levels as RV-A1B

**Figure 2:**
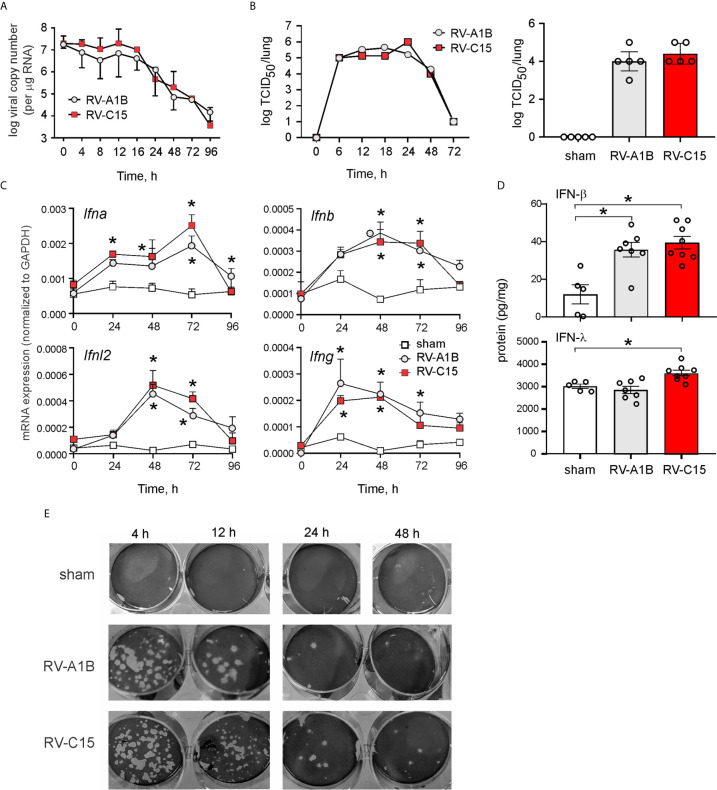
Viral RNA is detectable in the lungs of RV-C15 treated mice. **(A)** Female 8-10-week-old BALB/c mice were inoculated with 5 x 10^6^ ePFU of RV-C15 or RV-A1B by intranasal instillation and lungs were examined by RT-PCR for viral RNA at the indicated time points. Graph showing RV-C15 and RV-A1B copy number at the indicated time points up to day 4. Data are mean ± SEM, *n* = 2-16 mice/group from five different experiments. **(B)** Left panel. Time course of lung viral titers in mice infected with RV-C15 and RV-A1B (7 mice per virus). Viral titer was assessed by TCID_50_. Right panel. Group mean data from an additional 5 mice at the 48 hr time point are also shown. Median ± interquartile range, *n* = 5 mice per group from one experiment are shown. **(C)** Graphs showing IFN mRNA expression analysis at indicated time points. The level of gene expression for each sample was normalized to GAPDH. Data represent mean ± SEM, *n* = 6 mice in each group from two different experiments, *P<0.05 by two-way ANOVA. **(D)** Graphs showing IFN expression analysis 48 hr after infection. Data represent mean ± SEM, *n* = 7 mice in each group from one experiment, *P<0.05 by one-way ANOVA. **(E)** Plaque assays show live virus in the lungs of RV-A1B and RV-C15-infected mice, as evidence by plaque formation in HeLa-H1 and HeLa-E8 cells, respectively.

Female adult BALB/c mice were inoculated sham HeLa cell lysate, 5 x 10^6^ PFU RV-C15 or 5 x 10^6^ PFU RV-A1B (50 µl at 10^8^ PFU/ml) by intranasal instillation. Mice were sacrificed, and the lungs were analyzed by qPCR at 0-96 h after infection for the presence of viral RNA. Viral RNA levels peaked 12 h after inoculation (**Figure 2A**). RV-A1B and RV-C15 infectivity was assessed by homogenizing lungs from virus- or sham-inoculated mice, overlying this material onto confluent monolayers of HeLa-H1 or HeLa-E8 cells, and assessing viral infectivity. Clarified supernatants from lung homogenates of RV-C15-infected mice caused cytopathic effects in HeLa-E8 cells. Fifty percent tissue culture infectivity doses (TCID_50_) of RV-A1B and RV-C15 were determined by the Spearman-Karber method (38). Lung RV-A1B and RV-C15 titers peaked 24 h after inoculation (**Figure 2B**, left panel). There was no difference in lung viral titers 48 h after inoculation (**Figure 2B**, right panel). In addition, lungs from RV-A1B- and RV-C15-infected mice showed significant increases in IFN-α, β and l2 mRNA expression (**Figure 2C**) and IFN-β and IFN-l protein expression (**Figure 2D**), consistent with the presence of viral RNA. Lung homogenates of RV-C15-infected mice also formed small plaques on HeLa-E8 cells up to 48 h after infection, but homogenates from sham-infected mice did not (**Figure 2E**).

**Figure 3:**
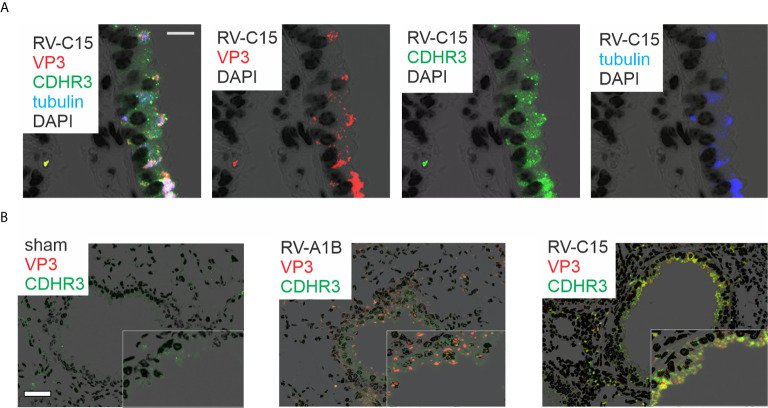
Colocalization of RV-C15 and CDHR3 in airway tissues of RV-C15-infected mice. **(A)** Airways from RV-C15-infected mice were stained with anti-VP3 (red), anti-acetyl α-tubulin (blue) and anti-CDHR3 (green). To stain mouse CDHR3, we identified a peptide (YQVEAFDPEDTSRN, human AAs 154-167) in the second extracellular calcium-binding domain identical in mouse and human. Polyclonal antiserum was generated and purified by affinity chromatography. Acetyl α-tubulin was localized to the epithelial cell apical surface. Colocalization is white. RV-C15 was localized to CDHR3+ ciliated airway epithelial cells. The white bar is 10 µ. **(B)** Airway sections from sham-, RV-A1B and RV-C15-infected mice stained for viral protein 3 (VP3, shown in orange/red) and CDHR3 (green). Colocalization is yellow. The white bar is 50 µ.

Lungs were also formalin-fixed and paraffin-embedded 24 h after exposure, and sections stained with fluorescent tagged anti-VP3, anti-CDHR3 and acetyl-α-tubulin. RV-C15 was localized to CDHR3+ ciliated airway epithelial cells (**Figure 3A**). Confocal microscopy using anti-YQVEAFDPEDTSRN showed colocalization of CDHR3 with RV-C15 but not RV-A1B (**Figure 3B**). However, it is important to note that this antibody did not block RV-C15 replication *in vitro* or *in vivo* (not shown).

### Infection of Cultured Mouse Airway Epithelial Cells With RV-C15

**Figure 4:**
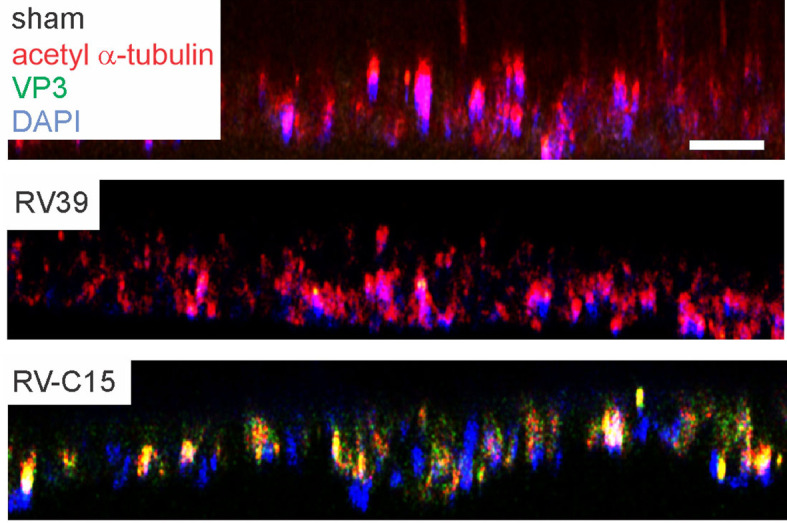
RV-C15 co-localizes with mouse epithelial cell acetyl-α-tubulin and induces expression of pro-inflammatory cytokines. Mouse airway epithelial cells were differentiated at air-liquid interface for 21 days. Cells were infected with sham HeLa cell lysate, RV-C15 or RV-A39 at MOI 2 and harvested 5 min after infection for immunofluorescence staining with anti-mouse acetyl α-tubulin (red) and anti-VP3 (green). Nuclei are stained with DAPI (blue). Confocal microscopy shows colocalization of cilia and RV-C15 (yellow). The white bar is 50 µ.

To confirm that RV-C15 infects mouse airway epithelial cells, we cultured differentiated mouse airway epithelial cells at air-liquid interface with sham, RV-C15 or RV-A39, a major group virus which does not infect mouse cells. Selected cell cultures were fixed and stained for AlexaFluor-conjugated anti-acetyl α-tubulin and anti-VP3. Cultures stained for acetylated tubulin indicating the presence of cilia (**Figure 4**, upper panel). Cultured inoculated with RV-A39 showed no VP3 present (middle panel). Cultures inoculated with RV-C15 showed colocalization of VP3 and acetyl α-tubulin, indicating infection of ciliated cells (lower panel).

### RV-C15 Induces Neutrophilic and Eosinophilic Inflammation and Expression of Type 2 Cytokines

**Figure 5:**
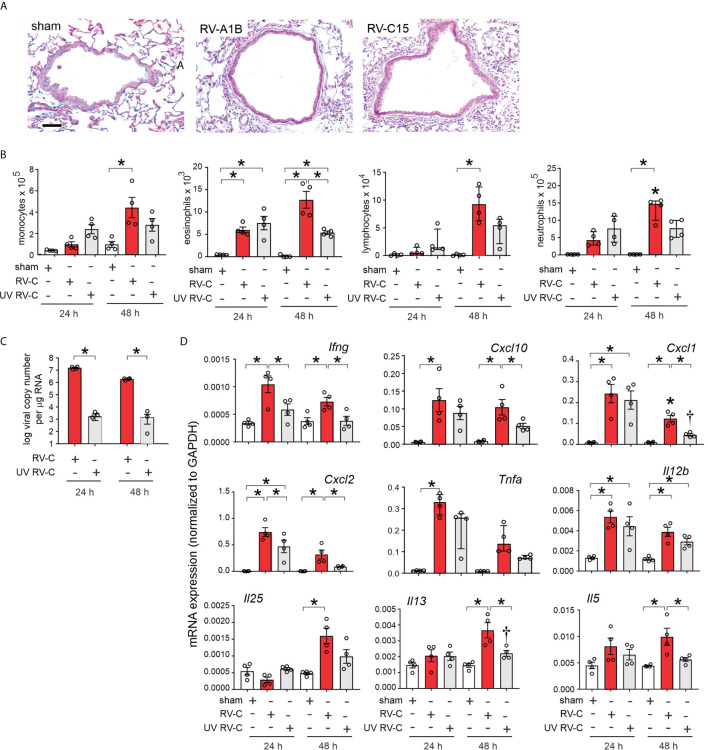
RV-C15 induces airway inflammation in naïve mice that is partially dependent on viral replication. Female 8-10-week-old BALB/c mice were treated with sham HeLa cell lysate, 5 x 10^6^ ePFU RV-C15 or UV-irradiated RV-C15. **(A)** Hematoxylin- and eosin-stained lung tissue. Bar = 50 µ. **(B)** Lungs were harvested 24 or 48 h after inoculation and processed for BAL inflammatory cell counts. **(C, D)** Twenty-four or 48 hr after inoculation, lungs were harvested for vRNA and mRNA expression. For mRNA, the level of gene expression for each sample was normalized to GAPDH. (For panels B-D, data are mean ± SEM except for lymphocytes, neutrophilis and *Tnfa, n* = 3-4 mice in each group from one experiment, *P < 0.05 by one-way ANOVA; for lymphocytes, neutrophils and *Tnfa*, data are median ± IQR, *P < 0.05 by Kruskal-Wallis test).

Forty-eight h post-inoculation, lungs were stained with hematoxylin and eosin. Sham-inoculated mice showed no inflammation (**Figure 5A**). However, RV-C15 exposed mice showed leukocyte infiltration around large airways, which was similar to RV-A1B. Next, we determined bronchoalveolar lavage inflammatory cell counts in RV-C15- and sham-infected mice 24 and 48 h after treatment. We also examined the effects of replication-deficient UV-irradiated virus. Selected RV-C15 preparations were irradiated with ultraviolet (UV) light at 1200 mJ/cm^2^ for 30 mi using a UVB CL-1000 cross-linker (39). Forty-eight h after inoculation, RV-C15-infected mice had significantly greater monocyte, neutrophil, lymphocyte and eosinophil recruitment into the bronchoalveolar fluid than sham-treated mice (**Figure 5B**). Mice infected with UV-irradiated virus showed significantly reduced viral copy number and fewer neutrophils, lymphocytes and eosinophils in the airways (**Figures 5B, C**).

Lungs were also harvested for mRNA expression 24 and 48 h after treatment, as measured by qPCR. Compared to sham treatment, RV-C15 infection significantly increased mRNA expression of IFN-γ, CXCL10, CXCL1, CXCL2, TNF-α, IL-12, IL-25, IL-13 and IL-5 (**Figure 5D**). UV irradiation significantly reduced RV-C15-induced IFN-γ, CXCL10, CXCL1, CXCL2, IL-13 and IL-5 transcript levels, and UV-irradiated virus failed to induce TNF-α or IL-25 mRNA expression.

### Comparison of RV-C15 and RV-A1B Responses

**Figure 6:**
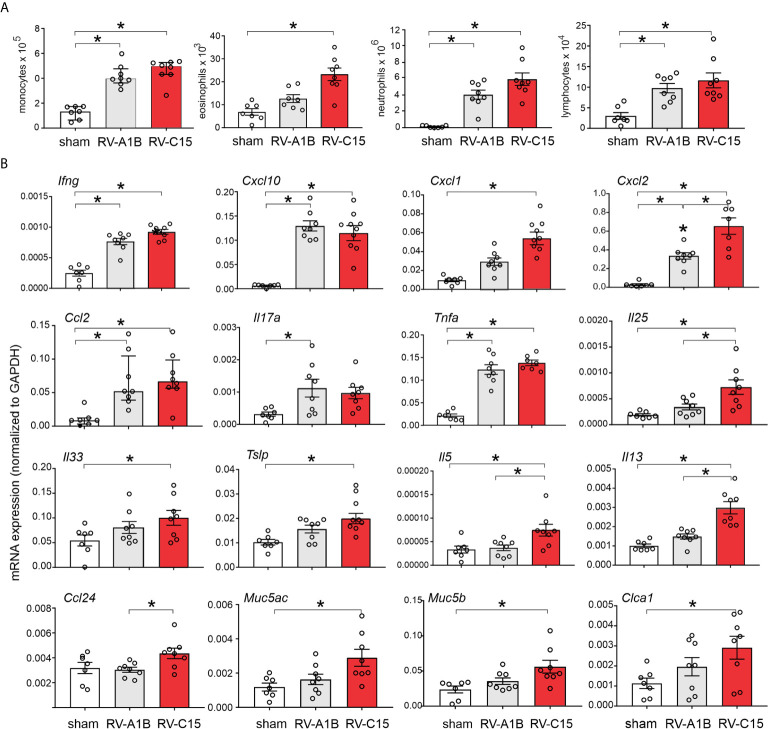
Comparison of RV-C15- and RV-A1B-induced airway inflammation in naïve mice. Female 8-10-week-old BALB/c mice were treated with sham HeLa cell lysate, 5 x 10^6^ ePFU RV-C15 or 5 x 10^6^ ePFU RV-A1B. **(A)** Lungs were harvested 48 hr after inoculation and processed for BAL inflammatory cell counts. **(B)** Forty-eight h after inoculation, lungs were harvested for mRNA expression. The level of gene expression for each sample was normalized to GAPDH. For panels **(A, B)** data are mean ± SEM except for monocytes and *Ccl2*, *n* = 7-8 mice in each group from two experiments, *P < 0.05 by one-way ANOVA; for monocytes and *Ccl2*, data are median ± IQR, *P < 0.05 by Kruskal-Wallis test).

**Figure 7:**
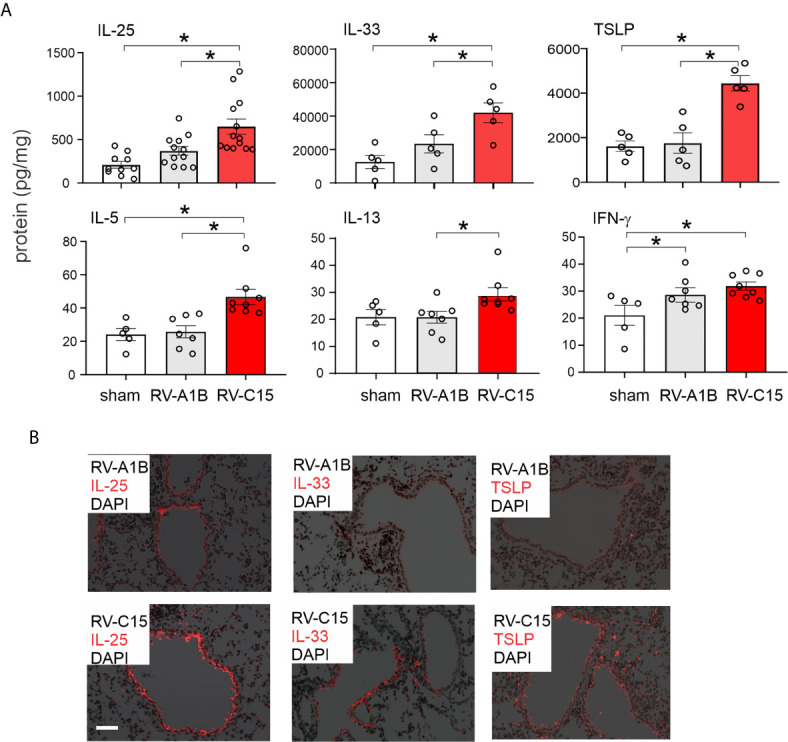
Comparison of RV-C15 and RV-A1B-induced cytokine expression in naïve mice. **(A)** Effects of RV-C15 and RV-A1B on cytokine expression measured by ELISA. Data are mean ± SEM, n = 5-12 mice in each group from one or two experiments, *P < 0.05 by one-way ANOVA. **(B)** Immunofluorescence images of mouse lungs infected with 5 x 10^6^ ePFU RV-C15 or 5 x 10^6^ ePFU RV-A1B. Staining for IL-25, IL-33 and TSLP is shown. Scale bar is 50 µm. These images are representative of four mice.

We compared lung inflammatory responses 48 h after infection with RV-C15 and RV-A1B, a minor group virus which infects mouse cells (29, 40) and constitutes a well-established model of RV infection (26, 27). As noted above, RV-C induced significant eosinophilic inflammation, whereas RV-1B did not (**Figure 6A**). Compared to RV-A1B, RV-C15 infection induced significantly higher mRNA expression of CXCL1, CXCL2, IL-5 and IL-13 (**Figure 6B**). Only RV-C15 induced significant mRNA expression of the IL-13-responsive genes Muc5ac, Muc5b and Gob5/Clca1. Finally, only RV-C15 significantly increased protein expression of the type 2 cytokines IL-5 and IL-13 (**Figure 7A**).

We also examined lungs for elaboration of IL-25, IL-33 and TSLP, innate cytokines that promote ILC2 expansion (41–47). Compared to RV-A1B, RV-C15 infection induced significantly higher mRNA expression of IL-25 (**Figure 6B**). Only RV-C15 significantly increased mRNA expression of IL-33 and TSLP. In addition, compared to RV-A1B, RV-C15 infection induced significantly higher protein expression of IL-25, IL-33 and TSLP (**Figure 7A**). Lungs were formalin-fixed and paraffin-embedded 2 days post-exposure, and sections stained for IL-25, IL-33 and TSLP immunofluorescence. Abundant staining for IL-25, IL-33 and TSLP was seen in the airways of mice inoculated with RV-C15 but not RV-A1B (**Figure 7B**). IL-25 and TSLP were localized to the airway epithelium whereas IL-33 was found in both airway epithelial and peribronchial cells.

### RV-C15 Inoculation Enhances mRNA Expression of Type 2 Cytokines, Mucus Genes and Airway Responsiveness in Allergen-Challenged Mice

**Figure 8:**
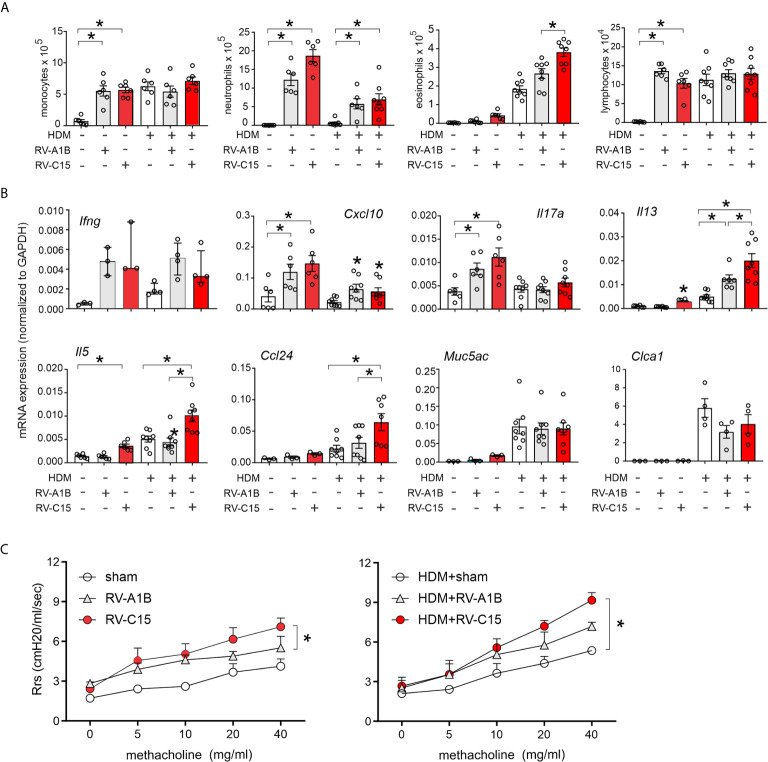
Comparison of RV-C15- and RV-A1B-induced airway inflammation in house dust mite-sensitized and -challenged mice. Female 8-12 week-old BALB/c mice were challenged with house dust mite (HDM) and treated one day after the last HDM treatment with sham HeLa cell lysate, 5 x 10^6^ ePFU RV-C15 or 5 x 10^6^ ePFU RV-A1B. Forty-eight hrs later lungs were harvested for BAL analysis and qPCR. A separate set of mice were similarly treated and were anesthetized and endotracheally intubated for measurement of airways responsiveness. **(A)** Graphs showing BAL cell counts. **(B)** Graphs showing qPCR analysis of lung mRNA expression. The level of gene expression for each sample was normalized to GAPDH. For panels **(A, B)**, data are mean ± SEM except for *Ifng*, *n* = 3-8 mice/group for 1-3 experiments, *p<0.05, one-way ANOVA; for *Ifng*, data are median ± IQR. **(C)** Airways methacholine responsiveness of the indicated treatment groups. Data are mean ± SEM of 3-4 mice/group from two experiments, *P < 0.05, two-way ANOVA.

RV-C15-induced respiratory illnesses have been associated with a previous history of asthma (3–6, 11, 13). We therefore determined the response to RV-C15 infection in mice with allergic airways disease and compared the responses with RV-A1B. As described previously (33), wild-type BALB/c mice were sensitized with house dust mite (HDM) and challenged with HDM 10 and 11 days after sensitization. Two days later, mice were inoculated with sham, RV-C15 or RV-A1B. Forty-eight h after infection, mice were sacrificed for bronchoalveolar lavage and lung mRNA determination or anesthetized and endotracheally intubated for measurement of methacholine responsiveness. As noted above, RV-C15 infection of naïve mice increased BAL monocytes, neutrophils, eosinophils and lymphocytes (**Figure 8A**), while inducing mRNA expression of IFN-γ, CXCL10, IL-17A, IL-13 and IL-5 (**Figure 8B**). HDM sensitization and challenge induced lung infiltration with monocytes, lymphocytes and eosinophils (**Figure 6A**), and lung mRNA expression of the eosinophil chemoattractant CCL24, the type 2 cytokines IL-5 and IL-13 and the mucus-related genes Muc5AC and Gob5 (**Figure 8B**).

Infection of HDM-treated mice with either RV-A1B or RV-C15 had additive effects on airway inflammation (**Figure 8A**). RV-A1B and RV-C15 each increased neutrophilic and eosinophilic inflammation; however, the increase in eosinophils was significantly greater in mice infected with RV-C15. In addition, RV-C15 infection of mice with allergic airways disease had additive effects on BAL lung IL-13, IL-5 and CCL24 expression, which were greater than induced by RV-A1B (**Figure 8B**). Thus, RV-C15 infection enhanced allergen-induced type 2 inflammation to a greater extent than RV-A1B. On the other hand, RV-C15-induced neutrophilic inflammation and mRNA expression of CXCL10 and IL-17 tended to be lower in allergen-challenged mice.

Next, we examined the effects of RV infection on the airway responsiveness in naïve and HDM-sensitized and -challenged mice. Increasing doses of nebulized methacholine were given by inhalation and respiratory system resistance measured. In naïve mice, RV-C15 increased airways responsiveness compared to RV-A1B (**Figure 8C**). In HDM-treated mice, only RV-C15 increased airway responsiveness compared to HDM alone.

### Potential Contribution of ILC2s to RV-C-Induced Airway Inflammation

**Figure 9:**
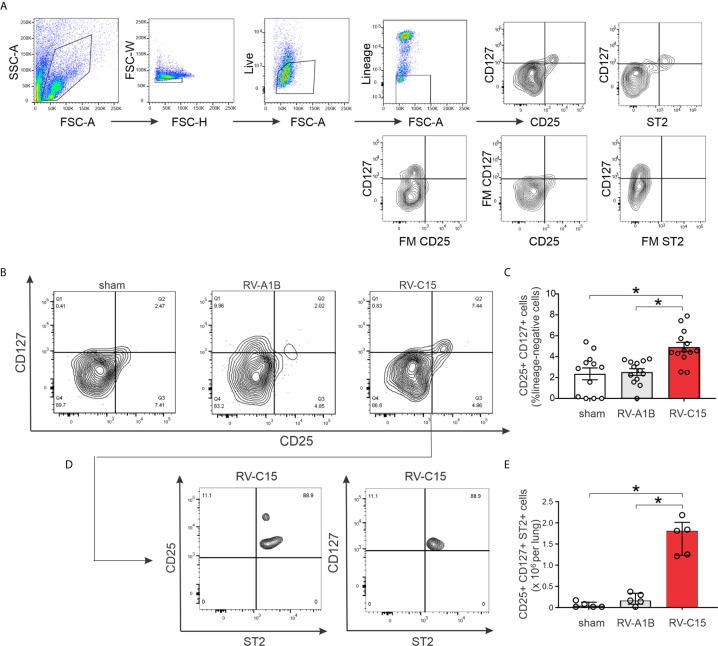
Flow cytometric analysis **(A–E)** for ILC2s was carried out in RV-A1B- and RV-C15-infected mice. For flow cytometry, ILC2s were identified as lineage-negative, CD25- and CD127-double positive cells **(B, C)** and lineage-negative, CD25+, CD127+, ST2+ cells **(D, E)**. For **(C)**, data are mean ± SEM, *n* = 12 mice in each group from three different experiments, *P < 0.05 by one-way ANOVA. For **(E)**, data are median ± IQR, *n* = 5 mice in each group from one experiment, *P < 0.05 by Kruskal-Wallis test.

Next, we examined lung ILC2s by flow cytometry. Cells were sorted for size, live/dead and surface markers for the various hematopoietic lineages (**Figure 9A**). Lungs from sham-treated mice showed a large number of lineage- CD25- CD127- cells, likely representing lung structural (epithelial and mesenchymal) cells. Two days after infection, RV-C15 infection was associated with a small but significant increase the number of lung lineage-negative, CD25-, CD127-double positive ILC2s (**Figures 9B, C**). There was no increase in the lungs of RV-A1B-infected mice. Identification of ILC2s was confirmed by co-staining with ST2, the IL-33 receptor (**Figures 9D, E**).

**Figure 10:**
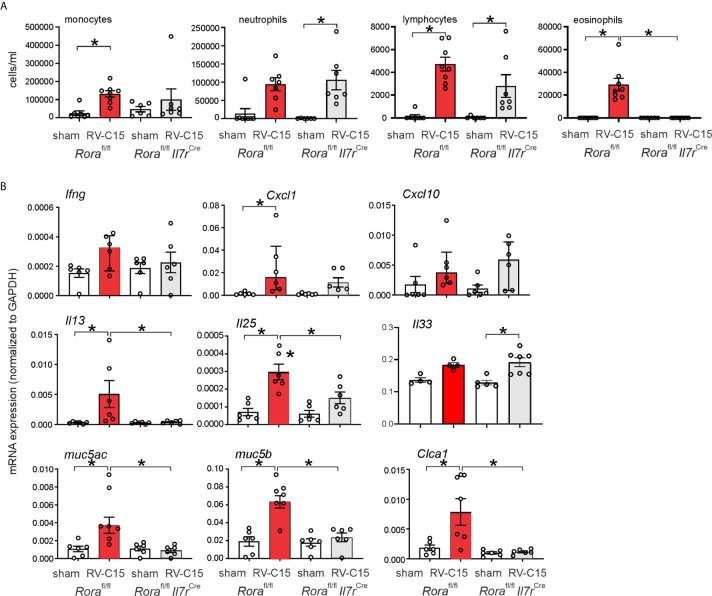
Requirement of ILC2s for RV-C15-induced airway inflammation and mRNA expression. **(A)** Rora^fl/fl^ Il7r^Cre^ mice infected with RV-C show lower eosinophils compared to Rora-^fl/fl^ littermates. Data are mean ± SEM, n = 6-8 mice/group from one experiment, *P < 0.05 by one-way ANOVA. **(B)** Rora^fl/fl^ Il7r^Cre^ mice infected with RV-C show lower *Il13, Muc5ac, Muc5b* and *Clca1* mRNA expression compared to Rora-^fl/fl^ littermates. Data are mean ± SEM except for IFN-γ and CXCL10, *n* = 6 mice/group from three different experiments, *P < 0.05 by one-way ANOVA; for *Ifng* and *Cxcl10*, data are median ± IQR.

We examined RV-C15-induced cytokine responses in ILC2-deficient *Rora* ^fl/fl^ *Il7r* ^Cre^ mice and *Rora* ^fl/fl^ littermates. We previously found that six day-old *Rora* ^fl/fl^ *Il7r* ^Cre^ mice fail to show ILC2 expansion after RV-A1B infection despite a small increase in viral load, demonstrating the effectiveness of this knockout (48). RV-C15 infection increased airway eosinophilic inflammation (**Figure 10A**) and mRNA expression of IL-13, Muc5ac and Muc5b in *Rora* ^fl/fl^ mice but not *Rora* ^fl/fl^ *Il7r* ^Cre^ littermates (**Figure 10B**). In contrast, RV-C15-induced neutrophils, lymphocytes and mRNA expression of IFN-γ and CXCL10 were preserved.

## Discussion

Despite increasing recognition of RV-C as a cause of severe asthma exacerbation, little is known about the pathogenesis of RV-C infections. To accomplish this, we infected BALB/c mice with RV-C15, and compared responses to our previously established RV-A1B model (27). Following intranasal inoculation with RV-C15, we isolated positive-strand viral RNA from the lungs of mice up to four days after exposure. We detected RV-C15 protein in airway epithelial cells. We also showed that lung homogenate from RV-C15-exposed mice 48 hr after infection causes cytopathic effects and plaque formation in HeLa-E8 cells. RV-C exposure induced a robust type I and type III interferon response which peaked 48-72 hrs after infection, evidence of viral replication (49). UV-irradiated virus had significantly reduced effects on airway inflammation and cytokine expression. RV-C15 exposure induced airway inflammation, as demonstrated by lung histology, increased BAL cells, and increased cytokine expression. Airways inflammation was accompanied by a functional state of hyperresponsiveness. Finally, RV-C15 but not RV-A39 infected ciliary-differentiated mouse airway epithelial cells cultured at air-liquid interface. Together, these data suggest that mouse lower airways may be infected with RV-C15. However, the steep reduction in viral RNA we observed in our model, similar to that previously observed for RV-A16 (26), minor group RV-A1B (26, 27) and enterovirus D68 (28), speaks against a substantial replicative infection.

In addition, we found that RV-C15 infection induced quantitatively and qualitatively different airway responses than RV-A1B. RV-C15-infected mice showed significantly higher CXCL1 and CXCL2 mRNA expression than RV-A1B-infected mice. In addition, only RV-C-infected mice showed increases in lung mRNA expression of IL-5, IL-13 and CCL24, indicating a type 2 inflammatory response. While at times the differences between RV-C15 and RV-A1B responses were small, RV-C-induced type 2 cytokine responses were sufficient to generate robust airway eosinophilia and mucous gene expression. We also found increased mRNA expression and peribronchial deposition of the innate cytokines IL-25, IL-33 and TSLP. This response is distinctly different from the response to RV-A1B or RV-A16, which have been shown by five different laboratories to be a classic type 1 antiviral response (26, 27, 50–55). Only Tbet-deficient mice show IL-13 expression after RV-A1B-infection (56). Similarly, after human experimental RV-A16 infection, only subjects with asthma, but not controls, show elevation of IL-5, IL-13, IL-25 or IL-33 (51, 57, 58). Similar differential IL-25 and TSLP expression has been noted in asthmatic epithelial cells (51, 59). This pattern of increased type 2 and innate cytokine expression could explain why infections with RV-C are more likely to occur in children with a history of asthma or who develop asthma (6, 10, 11, 13, 14).

The precise mechanism by which RV-C15 induces greater type 2 and innate cytokine expression than RV-A1B is unclear. A previous study examining the effects of various respiratory viruses on airway epithelial cell replication kinetics, cell tropism, tissue integrity, and cytokine secretion showed no differences between RV-C15 and other RVs (23). Similarly, we did not notice more cell loss in cultures infected with RV-C15 (data not shown). We speculate that differential innate cytokine expression following RV-C15 reflects the receptor-linked signaling pathways that establish the inflammatory response. In support of this hypothesis, differences in macrophage gene expression between two rhinovirus serotypes, RV-A16 and RV-A1A, have been traced to differential kinase and transcription factor phosphorylation following initial RV binding (60). Also in support of this hypothesis are the robust increases in cytokine expression we observed in mice treated with UV-irradiated RV-C15, more than we observed previously with RV-A1B (27). Responses to replication-deficient virus are likely to reflect receptor binding and endocytosis of virus, rather than later replication-dependent responses. Previous *in vitro* studies have also shown responses to replication-deficient RV (61–63), suggesting that binding and endocytosis of RV is sufficient, and viral replication unnecessary, for a subset of inflammatory responses, to be followed by a second set of replication-dependent responses.

We found colocalization of RV-C and acetyl α-tubulin, a microtubule protein that is enriched in the axonemes of most cilia. Thus, these data confirm that RV-C binds to ciliated airway epithelial cells (64). We found that CDHR3 preferentially colocalizes with RV-C15 compared to RV-A1B. Mouse CDHR3 is highly homologous to human CDHR3 and includes the N186 glycosylation site (19) and EC-1 domain (65) required and sufficient for RVC15 binding to the human protein. However, we were unable to block RV-C binding or replication with anti-CDHR3 (not shown). We are therefore unable to state with certainty that CDHR3 is the RV-C receptor in mice. Since structural analysis suggests that, similar to enterovirus-D68, RV-C15 binds to a sialic acid moiety of a CDHR3-bound glycan (18), it is conceivable that RV-C binds to other glycan-binding proteins. A recent study in human airway epithelial cells showed that, while CDHR3 knockdown blocks RV-C replication, it does not affect binding of RV-C (66), suggesting that a co-receptor is required for binding of the virus.

As noted above, RV-C15 infection induced airway expression of IL-25, IL-33 and TSLP, innate cytokines responsible for activation of IL-5- and IL-13-producing ILC2s expansion (41–47). IL-25 production was mostly limited to airway epithelial cells, whereas IL-33 was noted in epithelial and subepithelial cells. A similar pattern of IL-33 deposition was observed in children with steroid-resistant asthma (67). Accordingly, we found increased lineage-negative, ST2+, CD25- and CD127 ILC2s in the lungs of RV-C15-, but not RV-A1B-infected mice. ILC2 depletion blocked RV-C15-induced mRNA expression of IL-13 and the mucus-related genes Muc5ac and Muc5b. Previously we noted expansion of ILC2s in RV-A1B-infected 6 day-old mice but not mature mice (35). Lung ILC2 expansion has also been shown after influenza infection (68, 69) and allergen-induced airway inflammation (44–46, 70–72).

A limitation of our study is the transient nature of viral infection in our model. Species differences restrict viral replication, requiring a high inoculum. However, we have previously shown that infection with RV-A1B increases lung type 1 IFN and negative-strand viral RNA expression (27), markers of viral replication. In addition, MDA5 is required for RV-A1B-induced inflammatory responses (73), inferring a role for double-stranded RNA, a form of viral RNA which is only made during viral replication. Inhibition of inflammasome activation (55) and corticosteroids (74) each increase viral load in RV-infected mice, consistent with the notion that antiviral immunity plays a significant role in our model. In addition, we have observed differences in the inflammatory response to RV-A1B, RV-C15 and EV-D68 (28) which are qualitative in nature and resemble responses in human subjects. Taken together, these results suggest that while replication of human RV is minimal in mice, the resulting host-induced innate immune response and immunopathology is worthy of study. Indeed, replication-deficient viral vectors are a useful tool for studying the innate immune response to acute viral infection without ongoing cytopathic effects (75).

We conclude that RV-C15 exposure induces airways inflammation in mice, binding to ciliated airway epithelial cells. Compared to RV-A1B infection, the inflammatory response to RV-C15 is characterized by greater eosinophils, epithelial-derived innate cytokines and IL-13-producing ILC2s. Further characterization of this model, combined with studies of human subjects, will provide insight into the pathogenesis of rhinovirus C infections.

**Table 1:** Primer sequences for real-time PCR.

Gene	Primer sequences
CCL2	Forward:5’-GCTCTCTCTTCCTCCACCAC-3’
	Reverse:5’-GCGTTAACTGCATCTGGCT-3’
CCL24	Forward:5’-ACCTCCAGAACATGGCGGGC-3’
	Reverse:5’-AGATGCAACACGCGCAGGCT-3’
CXCL1	Forward:5’-TGCACCCAAACCGAAGTCAT-3’
	Reverse:5’-CAAGGGAGCTTCAGGGTCAAG-3’
CXCL2	Forward:5’-GCGCTGTCAATGCCTGAAG-3’
	Reverse:5’-CGTCACACTCAAGCTCTGGAT-3’
CXCL10	Forward:5’-GCTGCAACTGCATCCATATC-3’
	Reverse:5’-TTTCATCGTGGCAATGATCT-3’
GAPDH	Forward:5’-GTCGGTGTGAACGGATTTG-3’
	Reverse:5’GTCGTTGATGGCAACAATCTC-3’
GOB5	Forward:5’-CTGTCTTCCTCTTGATCCTCCA-3’
	Reverse:5’-CGTGGTCTATGGCGATGACG-3’
IFN-α1	Forward:5’-CCATCCCTGTCCTGAGTG-3’
	Reverse: 5’-CCATGCAGCAGATGAGTCCTT-3’
IFN-β	Forward:5’-CAGCCCTCTCCATCAACTATAAG-3’
	Reverse:5’-CCTGTAGGTGAGGTTGATCTTTC-3’
IFN-γ	Forward:5’-ACGCTACACACTGCATCTTGG-3’
	Reverse:5’-GTCACCATCCTTTTGCCAGTTC-3’
IL12b	Forward:5’-CTCCTGGTTTGCCATCGTTT-3’
	Reverse:5’-GGGAGTCCAGTCCACCTCTA-3’
IL13	Forward:5’-CCTGGCTCTTGCTTGCCTT-3’
	Reverse:5’-GGTCTTGTGTGATGTTGCTCA-3’
IL-17a	Forward:5’-GCCTGAGAGCTGCCCCTTCAC-3’
	Reverse:5’-GGCTGCCTGGCGGACAATCG-3’
IL-5	Forward:5’-CTCTGTTGACAAGCAATGAGACG-3’
	Reverse:5’-TCTTCAGTATGTCTAGCCCCTG-3’
IL-25	Forward:5’-ACAGGGACTTGAATCGGGTC-3’
	Reverse:5’-TGGTAAAGTGGGACGGAGTTG-3’
IL-33	Forward: 5’-GGCTGCATGCCAACGACAAGG-3’
	Reverse: 5’-AAGGCCTGTTCCGGAGGCGA-3’
Muc5ac	Forward:5’-AAAGACACCAGTAGTCACTCAGCAA-3
	Reverse:5’-CTGGGAAGTCAGTGTCAAACC-3’
Muc5b	Forward:5’-GAGCAGTGGCTATGTGAAAATCAG-3’
	Reverse:5’-CAGGGCGCTGTCTTCTTCAT-3’
TNF-α	Forward:5’-GCAGGTTCTGTCCCTTTCAC-3’
	Reverse:5’-GTCGCGGATCATGCTTTCTG-3’

## Data Availability Statement

The original contributions presented in the study are included in the article/**Supplementary Material**. Further inquiries can be directed to the corresponding author.

## Ethics Statement

The animal study was reviewed and approved by the Institutional Animal Care and Use Committee of University of Michigan and performed under National Institutes of Health guidelines.

## Author Contributions

CR performed experiments, analyzed the data, and drafted the manuscript. MYH performed experiments, analyzed data, and edited the manuscript. TI performed experiments and analyzed data, JL performed experiments. AG performed experiments and analyzed data. SJ performed experiments. CS performed experiments. JB performed experiments, analyzed data, and edited the manuscript. MBH supervised all aspects of the project, interpreted the data, and write the final draft of the manuscript. All authors contributed to the article and approved the submitted version.

## Funding

This work was supported NIH grants R01 HL134369 and R01 AI155444 (to MBH).

## Conflict of Interest

The authors declare that the research was conducted in the absence of any commercial or financial relationships that could be construed as a potential conflict of interest.
