# Supplementary material for: Rhinovirus C Infection Induces Type 2 Innate Lymphoid Cell Expansion and Eosinophilic Airway Inflammation
Source: Front Immunol. 2021 Apr 22;12:649520. doi: 10.3389/fimmu.2021.649520 (PMC8100319; doi:10.3389/fimmu.2021.649520)
Supplement: Supplementary file 1 [file DataSheet_1.docx]

**Supplemental Table 1. Primers for RV-C15 sequencing.** Primers were either designed using Primer BLAST with GenBank submission GU219984.1 or obtained from previously published work (Bochkov, Y. A., *et al*. Mutations in VP1 and 3A proteins improve binding and replication of rhinovirus C15 in HeLa-E8 cells. *Virology* 499: 350-360).

*Name Primer sequence Position Domain*

C15-5' END F TTA AAA CTG GGT ATA GGT TGT TC 1-23 5'UTR

RVC-16 -F1 CTC CCT TCC CGT AAC GTA GA 88-107 5'UTR

RVC-16 -F2 GGT GTC CGT GTT TCC TTT ATT C 538-559 5'UTR

RV-C15-F14 CCC AGA ATC CAC AAT AGA GAC 2455-2475 VP1

RV-C15-R2 GAA TAA AGG AAA CAC GGA CAC C 538-559 5'UTR

RV-C15-R13 GCT GGA GTT GTT GGT CCT CA 1184-1163 VP2

RVC-16 -F3 GGG CAA AGT GGT CAT CAA ATA C 1256-1277 VP2

RVC15-R3 GTA TTT GAT GAC CAC TTT GCC C 1277-1256 VP2

MLUI-F AACGCCAAGGCTTGCCAACG 1596-1615 VP3

RVC-16 -F4 CAG GTT TCC ACC CAA GTA AGA 1685-1700 VP3

BAMHI2-R TCATTTCTAGGGGCAGAACAAG 1847-1868 VP3

RVC-16 -F5 CTG TAG TGC TTG TCC CGA TAT G 2233-2250 VP3

RVC15-R5 CAT ATC GGG ACA AGC ACT ACA G 2250-2233 VP3

C15-VP1-F ATATTGGGTGCCATGGAGATTGGT 2414-2437 VP1

RV-C15-R14 GCG GAC CTA CCT AGG AAC AT 2541-2429 VP1

RVC-16 -F6 TGT TTG TTC CAC CAG GGA TAG 2700-2720 VP1

BLPI-R GACTCCCGGGGCCTGGAACATTGGTACTA 3159-3181 VP1

RVC-16 -F7 GCA TGG CGT GAT TGG CAT GA 3481-3500 2A

RVC16-R7 TCA TGC CAA TCA CGC CAT GC 3500-3481 2A

RV-C15-F15 TGT TGC TTT CAC TGA CCT ACG 3512-3532 2A

RV-C15-R15 GCT TAT GAT GCG CAC CAA CC 3628-3609 2B

RVC15-R8 CAA CAA ACA AAC TGG CTC GTG 4152-4132 2C

RVC-16-F8 TAG GCA CGA GCC AGT TTG TTT 4216-4233 2C

RV-C15-F16 GGC GAT TCT TTC TGG ATG CC 4482-4501 2C

QRV-C15F TGT CCT CTG TTG TGT GGT AAG 4615-4635 2C

RV-C15-R16 GCC TCA GCT GCT CTA CTA TTG 4793-4773 2C

QRV-C15R TGC CTC AGT TGC TCT ACT ATT G 4794-4773 2C

RV-C16-F9 GTG CTG AAC AGG GAT GGA TAG 4926-4646 3A

RVC-16-F10 GTA GAG GTT GGT GCT GTG ATA C 5389-5410 3C

RVC15-R10 GTA TCA CAG CAC CAA CCT CTA C 5410-5389 3C

RV-C15-F17 TCC AGG GGT GAA GGA ACC G 5788-5806 3D

RV-C15-R17 CCA CTT GTG TGT TAC CTT TGT AC 5891-5869 3D

RVC16-F11 GTG CAG GAT TCC CTT ATG TGA 6030-6048 3D

RVC-16 -F12 AGG TAT GCC CTC TGG TAT CT 6532-6531 3D

NDEI-F ATTATAGCATATGGTGATGATGTAGT 6647-72 3D

NDE1-R ACTACATCATCACCATATGCTATAAT 6672-47 3D

RV-C16-R1 ATT GAT CTA TCC ATT GTC TGT AC 7061-7039 3D

RV-C15-R18 GAA TTG ATC TAT CCA TTG CCT G 7063-7042 3D

3' UTR R GU219984 TATTATGATTCAAGATGAGTTTG 7111-089 3' UTR

**
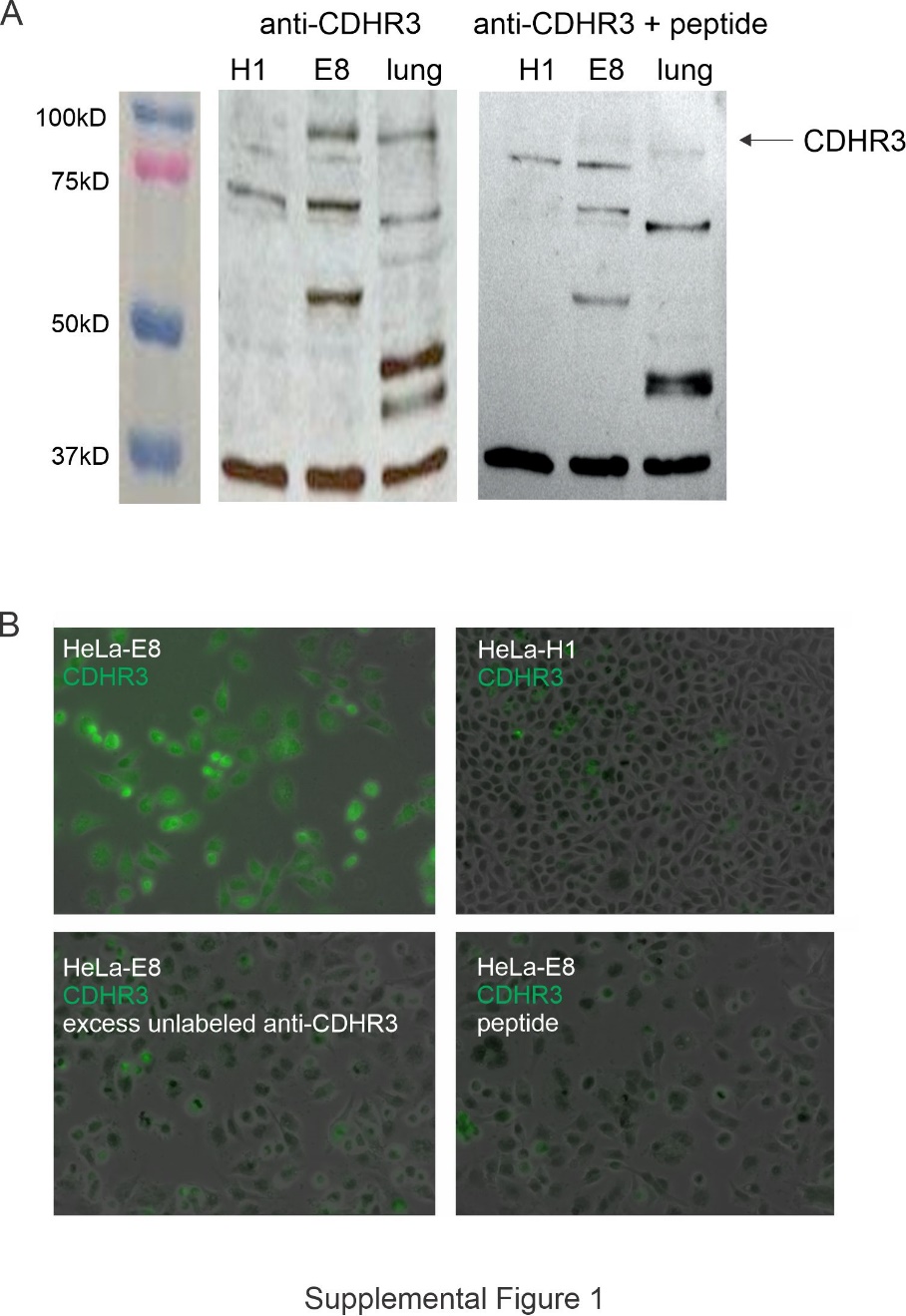
**

**Supplemental Figure 1.** Characterization of CDHR3 antibody. **A)** Anti-CDHR3 immunoblots of HeLa-H1 cells, HeLa-E8 cells and mouse lung in the absence or presence of YQVEAFDPEDTSRN peptide. **B)** HeLa-E8 cells and HeLa-H1 cells on poly-D-lysine-coated glass coverslips were stained with AF488-conjugated anti-CDHR3. Staining was blocked with addition of either unlabeled rabbit IgG or YQVEAFDPEDTSRN.
